# Transcriptional changes in response to ketamine ester-analogs SN 35210 and SN 35563 in the rat brain

**DOI:** 10.1186/s12864-019-5649-6

**Published:** 2019-04-11

**Authors:** Gregory M. Jacobson, Logan J. Voss, Anica Klockars, Steve Bird, Ivo Dimitrov, William A. Denny, Pawel K. Olszewski, James W. Sleigh, Martyn G. Harvey

**Affiliations:** 1 Faculty of Science and Engineering, The University of Waikato, Hillcrest, Hamilton, 3216 New Zealand; 2 Waikato District Health Board, Pembroke Street, Hamilton, 3204 New Zealand; 3 The University of Auckland, Grafton, Auckland, 1023 New Zealand

**Keywords:** Ketamine, Analgesia, Transcriptome, Glutamate, Potassium channels, Nociception

## Abstract

**Background:**

Ketamine ester analogs, SN 35210 and SN 35563, demonstrate different pharmacological profiles to ketamine in animal models. Both confer hypnosis with predictably rapid offset yet, paradoxically, SN35563 induces a prolonged anti-nociceptive state. To explore underlying mechanisms, broad transcriptome changes were measured and compared across four relevant target regions of the rat brain.

**Results:**

SN 35563 produced large-scale alteration of gene expression in the Basolateral Amygdala (BLA) and Paraventricular Nucleus of the Thalamus (PVT), in excess of 10x that induced by ketamine and SN 35210. A smaller and quantitatively similar number of gene changes were observed in the Insula (INS) and Nucleus Accumbens (ACB) for all three agents. In the BLA and PVT, SN 35563 caused enrichment for gene pathways related to the function and structure of glutamatergic synapses in respect to: release of neurotransmitter, configuration of postsynaptic AMPA receptors, and the underlying cytoskeletal scaffolding and alignment.

**Conclusion:**

The analgesic ketamine ester analog SN 35563 induces profound large-scale changes in gene expression in key pain-related brain regions reflecting its unique prolonged pharmacodynamic profile.

**Electronic supplementary material:**

The online version of this article (10.1186/s12864-019-5649-6) contains supplementary material, which is available to authorized users.

## Background

For more than 50 years ketamine has been used as an anaesthetic drug with an unparalleled safety profile. Latterly, ketamine’s identified analgesic and rapid-onset antidepressant properties have been incorporated into clinical practice. Regrettably, a growing market as a recreational drug of abuse is also apparent. Traditionally recognised as a non-competitive N-methyl-D-aspartate (NMDA) receptor antagonist, ketamine also binds with a broad range of other neuronal transporters and receptors, including opioid receptors, Dopamine D2 and monoamine transporters, serotonergic and cholinergic signalling systems, and non-NMDA glutamatergic receptors [1, 2]. Given ketamine’s complex pharmacologic profile it is perhaps unsurprising that the underlying mechanisms of its myriad of pharmacodynamic effects are yet to be fully elucidated.

Compared to ketamine, two recently developed ketamine esters – SN 35210 (subsequently referred to as R1) and SN 35563 (R5) – demonstrate a shorter recovery time and absent psychotomimesis following hypnosis [3]. In addition, it was shown that R5 treatment induced a profound analgesic effect that extended to at least one hour after the plasma level of the drug had completely disappeared due to rapid hydrolysis by tissue esterases. Because a related ketamine ester analogue with the same primary metabolite showed no such antinociceptive action, we are confident this analgesia is not mediated by an active metabolite.

While it is long held that the primary mode of action of ketamine is via the NMDA receptor, the drug also binds to other targets, including olfactory [4], acetylcholine [5], and pacemaker current (HCN1) [6] receptors, suggesting the possibility also for alternative modes of action for analogs of ketamine. One explanation for the mechanisms of prolonged analgesia exhibited by R5 is that it may induce key changes in early gene expression. It is known that ketamine and other NMDA receptor antagonists cause a variety of changes in expression of a significant number of genes [7, 8]. Therefore, we used mRNA sequencing to quantify gene expression changes induced in four key higher order pain-related regions of the rat brain by a 45 min infusion of ketamine and the related analogues. Based on this large-scale transcriptome analysis, we were able to define transcript classes most profoundly modified by each compound in each of the four brain regions. We used Reactome modelling in order to delineate potential intra- and inter-neuronal pathways affected by these drugs.

## Results

### Behavioural metrics

Infusion of all study drugs produced hypnosis characterised by Loss of Righting Reflex (LORR), and blunted responses to external stimuli. Dose to LORR was ketamine 27.9 (1.8) mg/kg, R1 55.1 (10.9) mg/kg, R5 77.1 (8.4) mg/kg. Total drug dosing to 45 min was ketamine 95.5 mg/kg, R1 229.8 (13.2) mg/kg, and R5 251.5 (9.3) mg/kg.

### Transcript sequencing

An average of 4.3 gigabases of sequence data was produced from each sample and after filtering this produced 30.1 (+/− 0.34) mb (Mean; SD) of clean reads for each sample. Processing by Cufflinks software produced a total transcript list of 21,602 +/− 148 (mean +/− SD) across the 16 samples, and this represents ~30X coverage (read number and data quality are included as Additional file 1).

### Differentially expressed genes

**Fig. 1:**
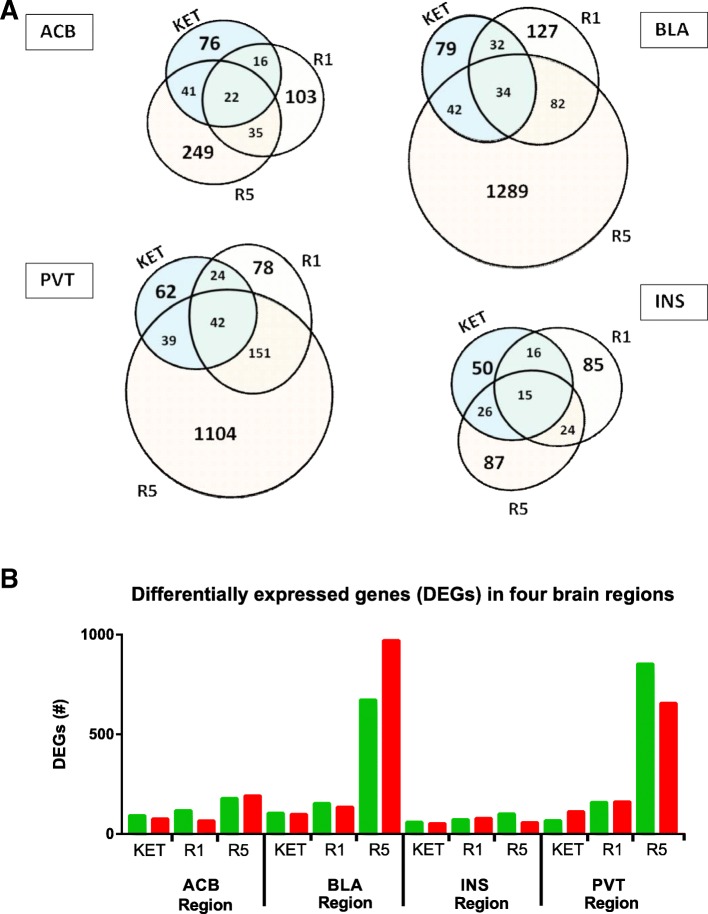
(**a**) Venn diagrams showing the scale of differentially expressed gene (DEGs) changes in the different brain regions (ACB, BLA, INS and PVT) examined; and (**b**) bar chart showing proportion of genes up- (green) and down-regulated (red) in each tissue

The lists of differentially expressed genes were sorted as up- and down-regulated transcripts for each brain region. The highest numbers of differentially expressed genes were found in the BLA and PVT from the R5-treated animals. A summary of the differentially expressed genes for each drug in different regions is shown in Fig. 1 (with detailed gene information, False Detection Rate [FDR] values, and fold change included as Additional file 2). The genes in each subset in the Venn diagrams in the figure are provided as another supplementary file (Additional file 3). Furthermore patterns of expression were confirmed for selected differentially expressed genes using quantitative PCR (qPCR) (Additional file 4).

### Reactome analysis reveals largest network changes are induced by R5 in BLA and PVT

**Fig. 2:**
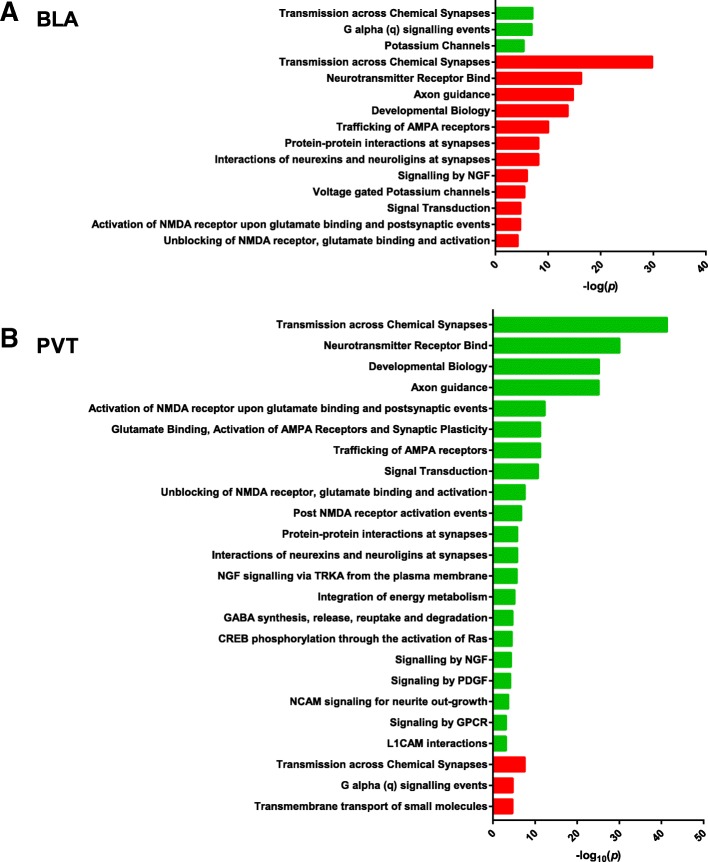
Reactome data from all differentially expressed genes in the 1% gene list from R5-treated animals for (**a**) BLA and (**b**) PVT by *p* value of enriched Reactome term. Upregulated enriched terms are in green while downregulated are in red

There were no enrichments for either ketamine or R1 drugs in any of the four regions examined, but large changes in the BLA and PVT in the R5-treated animals. The up- and down-regulated Reactome enrichments are summarised in Fig. 2. In some cases the same term appears as both up- and down-regulated and this reflects subsets of the analysed genes falling into these different grouping terms. The most prominent changes were seen in cellular functions that *controlled synaptic activity*, especially glutamatergic synapses. When separated into up- and down-regulated gene lists, the R5-induced changes in the BLA were broadly opposite to the direction of changes seen in the PVT.

### Interaction network analysis: features of R5-induced gene networks

**Fig. 3:**
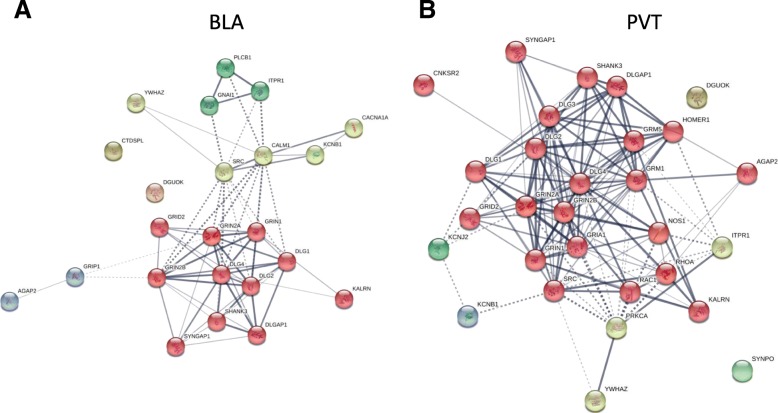
Combined STRING graphic of all enriched interactions for R5 in: (**a**) BLA and (**b**) PVT. Up- and down-regulated gene lists were combined. Each node represents genes whose expression levels were altered by treatment with R5. Links between nodes represent the strength of association between gene changes

In agreement with the aforementioned Reactome analysis, the ketamine and R1 treatments did not result in any enrichment for interactions in the examined regions. In contrast, significant enrichment was seen for R5, as shown in Table 1 (top 10 most enriched). A STRING diagram shows the enriched terms for the BLA and PVT with R5 treatment (Fig. 3).

**Table 1:** Enrichment of differentially expressed genes (DEGs) for known interactions (“interactome”) for each region and drug combination. The most enriched (up to 10) are shown for each brain region/drug combination

Region	# interactions (*p* < 0.5)	Top interactions	*p* value
ACB	4	YWHAZ interactions	1.69E-06
		IPPK interactions	2.75E-06
		MAP2K4 interactions	4.12E-06
		NANOG interactions	4.31E-06
BLA	23	DLG4 interactions	4.32E-23
		DLGAP1 interactions	2.05E-19
		SYNGAP1 interactions	5.91E-17
		AGAP2 interactions	4.43E-16
		SHANK3 interactions	1.87E-15
		GRIN2B interactions	2.49E-12
		GRIN1 interactions	9.86E-11
		GRID2 interactions	1.70E-09
		DLG1 interactions	2.44E-09
		YWHAZ interactions	5.69E-09
INS	1	NTRK1 interactions	9.35E-07
PVT	29	DLGAP1 interactions	2.26E-22
		DLG4 interactions	8.56E-22
		AGAP2 interactions	3.13E-20
		SYNGAP1 interactions	1.00E-19
		SHANK3 interactions	5.25E-19
		GRIN2B interactions	9.05E-15
		GRIN1 interactions	9.32E-14
		GRIN2A interactions	4.19E-13
		YWHAZ interactions	5.32E-10
		KCNB1 interactions	5.83E-10

**Fig. 4:**
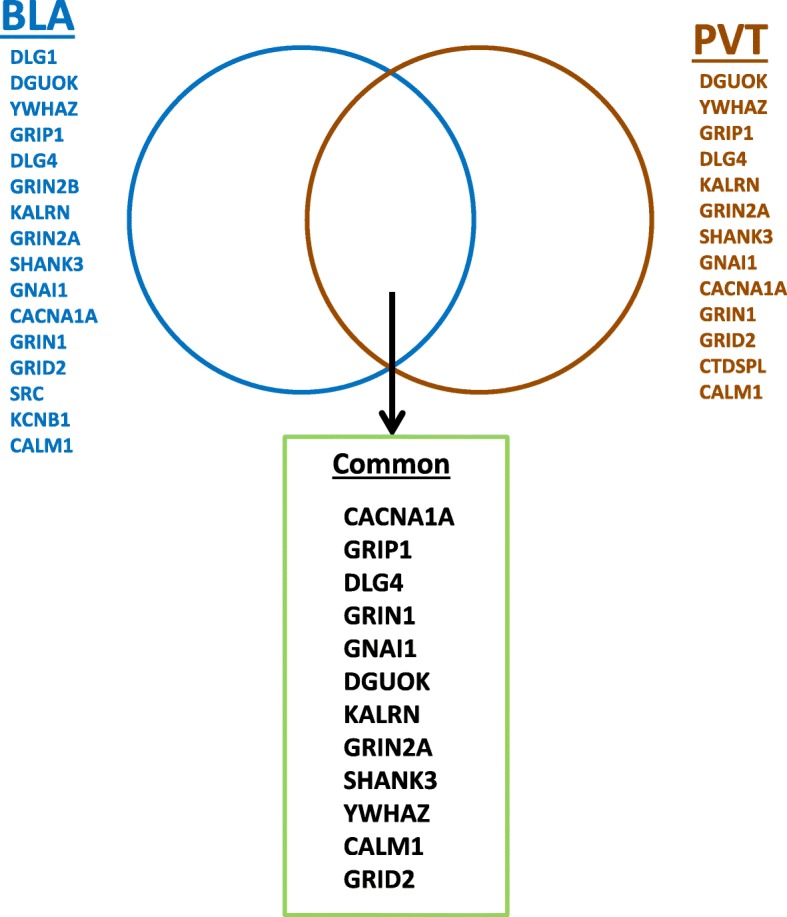
Independent and shared interaction terms for R5 in BLA and PVT regions for terms enriched in Interactome analysis, but not showing differential expression in response to drug treatments

It has been shown previously that for a given drug treatment, the expression levels of the drug binding targets themselves are very unlikely to be altered [9]. Instead, networked proteins downstream to the target are more likely to show dysregulated expression. Applying this rationale, we identified significantly enriched interaction terms that were not differentially expressed (Fig. 4, Table 2).

**Table 2:** Known functions of candidate R5 target proteins (“common” list). Descriptions are from UniProt database (www.uniprot.org)

Common terms	Description
CACNA1A *Calcium Voltage-Gated Channel Subunit Alpha1 A*	Voltage-sensitive calcium channel involved in neurotransmitter release
GRIP1 *Glutamate Receptor Interacting Protein 1*	Scaffold for multi-protein signalling complexes and mediator of trafficking of its binding partners, related to glutamate receptors.
DLG4 *Discs Large MAGUK Scaffold Protein 4*	Guanylate kinase (MAGUK) family member. Mediator of post-synaptic events; including plasticity via by interaction with cytoplasmic tail of NMDA receptor (NMDR), DLG2 and shaker type potassium channels following ligand binding.
GRIN1 *Glutamate Ionotropic Receptor NMDA Type Subunit 1*	NMDA receptor subunit associated with synaptic plasticity
GNAI1 *Guanine Nucleotide-Binding Protein G(I) Subunit Alpha-1*	Signal transduction downstream of G-protein coupled receptor
DGUOK *Deoxyguanosine Kinase*	Nucleoside kinase activity in mitochondria
KALRN *Kalirin RhoGEF Kinase*	Signal transduction of pathways for neuronal shape, growth and plasticity mainly via effects on actin cytoskeleton
GRIN2A *Glutamate Ionotropic Receptor NMDA Type Subunit 2A*	NMDA receptor subunit associated with long-term potentiation and efficiency of transmission
SHANK3 *SH3 And Multiple Ankyrin Repeat Domains 3.*	Scaffold protein between membrane proteins and cytoskeleton. Involved in signal transduction
YWHAZ *Tyrosine 3-Monooxygenase/Tryptophan 5-Monooxygenase Activation Protein Zeta*	Regulation of many signalling pathways via binding to phosphoserine containing proteins
CALM1 *Calmodulin 1*	Involved in control of function of many proteins and calcium-dependent inactivation of CACNA1C

## Discussion

### Overview of findings

Ketamine is well-known for its ability to induce a state of dissociative anaesthesia and profound analgesia. Reports of rapid-onset antidepressant action extending far beyond the pharmacokinetic time course of the drug have further increased clinical applications, as well as stoking academic interest in the underlying mechanisms of action for ketamine. The novel ketamine ester analogues R1 and R5 have been developed from ketamine with the specific goal of achieving fast offset hypnosis and absent psychomimetic properties through rapid tissue mediated hydrolysis to inactive metabolites. However, while both analogues confer accelerated arousal, only R5 exhibits a prolonged anti-nociceptive action that persists for up to 90 mins following cessation of drug administration.

In the present experiment we utilise drug-induced gene expression profiling to explore the disparate neuro-behavioural effects of these drugs. In particular, we were interested in the potential of R5 to induce identifiable transcriptome-level changes in dedicated pain-related brain regions. In summary, we found that the compounds all altered individual gene expression for about 50–100 genes in all the brain regions examined, but in addition R5 profoundly altered the expression of many more genes in specific brain regions.

Ketamine and R1 induced broadly comparable changes both in terms of the number and scope of gene perturbations. Literature reports of ketamine-induced gene alterations in brain tissue demonstrate significant heterogeneity. Ficek et al. [10] identified 52 gene transcripts (including for Dusp1, Per1 and Fkbp5 genes) with altered expression in mouse Striatum and Hippocampus following ketamine administration. Functional linkage was reported with mitogen-activated protein kinase (MAPK), Il-6, and insulin signalling pathways with expression differing throughout the eight hours sampling interval. In a study exploring the role of ketamine and sleep deprivation in a mouse model Orozco-Solis et al. 2017, have shown alterations in expression levels of 1149 genes in the Anterior Cingulate Cortex using a threshold of *p* < 0.05 [8]. For the 64 transcripts that were in both the sleep and ketamine datasets, the related ontologic categories included entrainment of the circadian clock, regulation of dentritic morphogenesis, ribosome function, nucleic acid binding, and cellular metabolic processing. Lack of commonality in reported gene alterations following ketamine exposure likely reflect both dose and time dependent factors in employed models, in addition to variation in sites of brain sampling.

In our work we have also observed significant variability in ketamine-induced gene expression across the different interrogated brain regions. Indeed, of all differentially expressed genes only 49 were commonly expressed in two brain regions following ketamine treatment, 29 in three brain regions, and eight (HSD17B11, NCAPD3, SLITRK5, KCNG1, NUP205, NCAPD3, SREK1, CYTH3) differentially expressed across all four brain regions examined. Clearly, in addition to exposure and sampling intervals, ketamine-induced transcriptome signatures demonstrate significant regional variability.

In contrast, R5 altered expression in about ten times the number of genes in regions of the brain linked to high-level analgesia networks – the BLA and PVT [11–15]. This was supported by the gene transcription network analyses, which showed no significant enrichment in ketamine and R1 animals. The main effects of R5 were on the expression of various genes controlling the function and structure of glutamatergic synapses; both as regards release of neurotransmitter, configuration of postsynaptic AMPA receptor, and the underlying cytoskeletal scaffolding and alignment.

### R5 analog causes alterations in postsynaptic density-associated gene networks

In animals treated with R5, in the BLA and PVT there was a strong enrichment for interactions associated with postsynaptic density Discs Large MAGUK Scaffold Protein 4 (PSD95), which is encoded by the DLG4 gene and has a central role in NMDAR trafficking, membrane-targeting and internalization [16]. YWHAZ is involved in the regulation of actin filament dynamics and is differentially expressed after glutamate exposure in hippocampal slice cultures [17]. SHANK3 is involved in NMDA receptor tethering and dendritic spine rearrangement and has been identified as a regulator of the anti-depressive properties of ketamine [18].

These proteins are components of the postsynaptic density network that connects many membrane proteins, including the excitatory neurotransmitter receptors NMDA, AMPA and mGlut, to the actin cytoskeleton. The association of ketamine with dysregulation of genes involved in postsynaptic density has been reported previously [19–21]; and Yang and colleagues [22] observed increased dendritic filopodia after 4 h of anaesthesia with ketamine-xylazine. Furthermore, the anaesthetic effect of halothane is enhanced in mice following disruption to PSD95-PDZ2 with intraperitoneal injection of a Tat-PSD95-PDZ2 fusion protein [23]. These studies highlight that cytoskeletal protein networks represent a novel anaesthetic target, disruption of which could have functional implications. Changes in the expression of scaffolding protein components of this network are associated with synaptic plasticity through regulation of receptor localisation and distribution [24], and interactions between PSD95 and NMDR subunits GRIN1 and GRIN2, and between PSD93 and AMPAR are particularly important [25]. The postsynaptic density network also mediates the efficiency of neurotransmission possibly through arrangement of so-called ‘nanocolumns’, aligning pre-synaptic neuron regions rich in secreted neurotransmitter-containing vesicles with the receptors of the postsynaptic neuron [26].

There is also accumulating evidence of the importance of AMPA receptors in analgesic mechanisms associated with anaesthetic and non-anaesthetic drugs – such as xenon [27], halothane [28], and the AMPA antagonist Tezampel [29]. The amygdala is known to be associated with the emotional component of the pain experience [30, 31], and is linked with the paraventricular thalamus [32] in a circuit that modulates the endogenous descending anti-nociceptive pathways [33]. Even within the amygdala itself, there are intricately linked excitatory and inhibitory modules. Clearly the mechanistic details of how R5 alters the balance will require specific future experiments.

Lisek et al. [21] studied the effects of ketamine on psychosis and showed that ketamine increases synaptic glutamate release in the cortex and striatum. This was associated with an increased expression of glutamate transporters, VGLUT1 (SLC17A7) and VGLUT2 (SLC17A6), and a decreased expression of membrane glutamate reuptake pump excitatory amino acid transporter 2 (EAAT2) (a.k.a. solute carrier family 1 member 2: SLC1A2). In our experiment, no differential expression in these genes was seen with ketamine treatment in any tissue. However, in R5-treated animals, contrary changes in VGLUT gene expression were seen in the BLA (VGLUT1 down; VGLUT2 up) and PVT (VGLUT1 up; VGLUT2 down). Neither region however showed changes in EAAT2/SLC1A2 expression. There is a possibility that these differences may explain some pharmacodynamic features of R5, such as reduced behavioural changes consistent with diminished post-anaesthetic psychosis (Harvey, unpublished observations).

## Conclusion

We have demonstrated, using transcriptomic analysis with various bioinformatics tools, that the antinociceptive action of R5 is associated with large-scale early transcriptome changes in the key pain-related brain regions, the BLA and PVT. Predominance of dysregulated glutaminergic signalling machinery suggests that it may serve as a functionally relevant binding target in R5-mediated analgesia. These will be explored later for relevance in the novel effects of the analogues such as prolonged analgesia and memory effects on pain perception.

## Methods

### Drugs and animals

Ketamine (Hospira Australia Ltd., VIC, Australia) was sourced from a commercial supplier. Ketamine analogues R1 and R5 (R1: SN35210 = racemic methyl 4-((1-(2-chlorophenyl)-2-oxocyclohexyl)amino)pentanoate hydrochloride and R5: SN35563 = racemic isopropyl 3-((1-(2-chlorophenyl)-2-oxocyclohexyl)amino)propanoatehydrochloride were synthesized according to a previously reported procedure [3] by the Auckland Cancer Society Research Centre Laboratory (University of Auckland, New Zealand).

Ethical approval for animal experimentation was obtained from the University of Waikato Animal Ethics Committee (University of Waikato, Hamilton, New Zealand). Adult female Sprague-Dawley rats (280–370 g) obtained from breeding stock from the University of Waikato small animal unit were randomised (random number generation) into four groups of five animals for the experiments. Rats, housed in standard Plexiglas cages under constant temperature (21 °C) and a 12:12 light/dark cycle (lights on at 07:00), were allowed ad libitum access to standard laboratory chow and tap water until the day of the experiment. All experiments were conducted during the first half of the light phase.

### Drug treatments

Sedative infusions (defined by continuous Loss of Righting Reflex [LORR]) for ketamine, R1 and R5, and negative control were undertaken following venous cannulation of the marginal tail vein. Ketamine-treated animals (*n* = 5) received intravenous ketamine (10 mg/mL) initially at 20 mg/kg/min until LORR, and thereafter at 2 mg/kg/min to 45 mins. R1-treated animals (n = 5) received intravenous SN 35210 (10 mg/mL) initially at 20 mg/kg/min until LORR, and thereafter at 4 mg/kg/min to 45 mins. R5-treated animals (n = 5) received intravenous SN 35563 (10 mg/mL) initially at 20 mg/kg/min until LORR, and thereafter at 4 mg/kg/min to 45 mins. Control animals (*n* = 5) received continuous infusion of 0.9% saline solution at 0.4 mL/kg/min for 45 mins.

### Tissue collection and RNA isolation

After 45 mins, drug infusions were halted and animals immediately euthanized by decapitation. Brains were dissected and the Nucleus Accumbens (ACB), Basolateral Amygdala (BLA), Paraventricular nucleus of the Thalamus (PVT) and Insula (INS) collected based on the coordinates from the Paxinos and Watson brain atlas. The tissue from each region was divided in a bilateral fashion, i.e., one side was placed into one tube as a pooled sample for each treatment group and the other side placed in separate tubes corresponding to a given brain site in each individual animal. The tubes contained RNAlater™ (Thermo Fisher Scientific, NZ). After overnight incubation at 4 °C in RNAlater, the samples were frozen at − 20 °C. The pooled samples (consisting of 16 tubes - control and three different drug treatments - namely ketamine, R1 or R5 - for each of four brain regions) were submitted for transcriptome sequencing (BGI, Shenzhen, Guangdong, China). For longer term storage the tubes containing ACB, BLA and INS of each individual animal were stored in RNAlater™ at − 80 °C for later qPCR analysis.

### Next generation sequencing, filtering and mapping

The processing and analysis in this and the next section (splicing variation analysis) were carried out by a commercial service (BGI, Shenzhen, Guangdong, China). Briefly, total RNA was extracted from the pooled brain tissue for each region and mRNA enriched using Oligo(dT) magnetic beads. cDNA was made using random hexamers, a size selection performed and amplification carried out by a PCR step. Transcriptome sequencing and basic filtering was on an Illumina Hiseq platform. After sequencing, reads of low-quality, containing adaptor sequences and those with a high content of unknown bases, were removed. Read data from each sample were mapped to a reference genome using a bioinformatic approach including Stringtie [34] to reconstruct transcripts and the Cuffcompare module of Cufflinks [35]. Novel transcripts were merged with reference transcript sequences and clean reads mapped using Bowtie2 [36]. Then gene expression level was determined using RSEM [37].

### Data interpretation and bioinformatics

In this study we applied a number of tools to gain insight into the functional significance of the expression data. In the first instance, genes that showed a greater than a two-fold change in drug-treated vs. control animals, and were also included in the top 1% as ranked by FDR level, were classified as *differentially expressed genes* (giving a final FDR cut-off of *p* < 0.0005). This is obviously a conservative classification, aimed to reduce the chance of random changes in gene expression being falsely attributed to a drug effect. Because of the low power, we have probably underestimated the actual drug effects on gene expression.

Subsequently, the question arose as to whether the changes in gene expression were occurring in isolated genes, or whether there was a consistent pattern of altered gene expression linked to certain functional pathways within a cell. The latter implies greater functional impact. Therefore, the list of differentially expressed genes was queried against public databases of known gene/protein cellular functions to reveal whether the list had over-representation (was *enriched*) for associations with a particular functional term, e.g. “*transmission across chemical synapses*”. Two databases were interrogated, namely the Reactome and the Interactome.

### Reactome

The Reactome is a curated database that summarizes both gene- and protein- functional associations. Reactome analysis was carried out by searching gene lists using rat genome database IDs corresponding to each condition and brain region using the Panther ontology database [38] (with Bonferroni FDR applied). This produced an output of Reactome terms that were enriched in the lists. These same enrichments were also visualised using gene symbol annotations at the Reactome database [39].

### Interaction/interactome analysis

The Interactome is the collection of all known and publicly available protein-protein associations for the proteins that would be produced from a submitted list of genes. ‘Interaction analysis’ thereby is a test of whether a particular gene list has significantly more terms associated with a particular known functional cellular interaction than would be expected by chance. Differentially expressed genes were searched by gene symbol using the TOPPGENE database [40]. All interactions with a FDR < 0.05 from the 1% of 0.05 FDR gene lists were considered. To produce the short list of possible binding targets for the drugs, we applied a previously described approach that assumes that gene expression of drug target proteins is not likely to be altered by treatment with that drug [9]. Our approach differed slightly in that we did not include Search Tool for InteracTions of CHemicals (STITCH) [41] database analysis as that database has no data on the analogue drugs. The interaction terms were filtered using this prediction of no change in expression of actual targets. The highest scoring enriched networks for each brain region/drug combination from this filtered list were profiled using the STRING database [42]. For STRING, all terms for each region were clustered by Markov Cluster Algorithm clustering (MCL) by inflation parameter of 6 with confidence setting at 0.4 (medium).

## Additional files

Additional file 1: QC sequencing. Quality control and summary statistics data for RNA sequencing experiment. (PDF 181 kb)

Additional file 2: Detailed gene lists. Excel spreadsheet with worksheets showing all differentially expressed genes found in each sample and within the false detection rate cut-off used. (XLSX 591 kb)

Additional file 3: DEGs in Venn diagram. Lists of DEGs found in all Venn diagram subsets, showing unique and shared genes between control (vehicle) and drug treatments. (XLSX 89 kb)

Additional file 4: qPCR data. qPCR data for tested changes in gene expression. (DOCX 13 kb)

## Abbreviations

ACB: Nucleus Accumbens
AMPA: α-amino-3-hydroxy-5-methyl-4-isoxazolepropionic acid
AMPAR: Glutamate Ionotropic Receptor AMPA Type
BLA: Basolateral Amygdala (BLA), cDNA - complement DNA
CYTH3: Cytohesin 3
DLG4/PSD95: Discs Large MAGUK Scaffold Protein 4
DNA: Dideoxyribonucleic acid
Dusp1: Dual Specificity Phosphatase 1
EAAT2: Excitatory Amino Acid Transporter 2
FDR: False Detection Rate
Fkbp5: FK506 Binding Protein 5
GRIN1/2: Glutamate Ionotropic Receptor NMDA Type Subunit 1 / 2
HSD17B11: Hydroxysteroid 17-Beta Dehydrogenase 11
IL-6: Interleukin 6
INS: Insula
KCNG1: Potassium Voltage-Gated Channel Modifier Subfamily G Member 1
LORR: Loss Of Righting Reflex
MAPK: Mitogen-activated protein kinase
MCL: Markov Cluster Algorithm clustering
mGlut: Metabotropic glutamate receptor
mRNA: Messenger RNA
NCAPD3: Non-SMC Condensin II Complex Subunit D3
NCAPD3: Non-SMC Condensin II Complex Subunit D3
NMDAR: N-methyl-D-aspartate Receptor
NUP205: Nucleoporin 205
Per1: Period Circadian Regulator 1
PSD93: Discs Large MAGUK Scaffold Protein 3
PVT: Paraventricular Nucleus of the Thalamus
qPCR: Quantitative PCR
R1: SN 35210
R5: SN 35563
SHANK3: SH3 And Multiple Ankyrin Repeat Domains 3
SLC1A2: Solute Carrier Family 1 Member 2
SLITRK5: SLIT And NTRK Like Family Member 5
SREK1: Splicing Regulatory Glutamic Acid And Lysine Rich Protein 1
STRING: Search Tool for recurring Instances of Neighbouring Genes
Tat-PSD95-PDZ2: Postsynaptic density protein-95 Discs large-Zonula occludens domain 2
TOPPGENE: Transcriptome, Ontology, Phenotype, Proteome, and Pharmacome annotations based gene list functional enrichment analysis
VGLUT1/SLC17A7: Solute Carrier Family 17 Member 7
VGLUT2/SLC17A6: Solute Carrier Family 17 Member 6
YWHAZ: Tyrosine 3-Monooxygenase/Tryptophan 5-Monooxygenase Activation Protein Zeta
