# Supplementary material for: Transcriptional changes in response to ketamine ester-analogs SN 35210 and SN 35563 in the rat brain
Source: BMC Genomics. 2019 Apr 11;20:281. doi: 10.1186/s12864-019-5649-6 (PMC6458767; doi:10.1186/s12864-019-5649-6)
Supplement: Supplementary file 1 — QC sequencing. Quality control and summary statistics data for RNA sequencing experiment. (PDF 181 kb) [file 12864_2019_5649_MOESM1_ESM.pdf]

Sample	Total Clean Reads	Total Mapping Ratio	Total Raw Reads (Mb)	Total Clean Reads(Mb)	Total Clean Bases(Gb)	Clean Reads Q20 (%)	Clean Reads Q30 (%)	Clean Reads Ratio (%)
**ACB_Control**	30022508	85.08%	37.25	30.02	4.5	98.4	95.31	80.6
**ACB_KETAMINE**	30372596	85.29%	37.25	30.37	4.56	98.42	95.35	81.54
**ACB_R1**	30209912	85.27%	37.25	30.21	4.53	98.44	95.38	81.11
**ACB_R5**	30468892	85.71%	37.25	30.47	4.57	98.4	95.25	81.8
**BLA_Control**	29432304	84.74%	35.63	29.43	4.41	98.46	95.46	82.61
**BLA_KETAMINE**	30190824	84.96%	37.25	30.19	4.53	98.39	95.31	81.06
**BLA_R1**	29948020	85.87%	35.63	29.95	4.49	98.36	95.21	84.06
**BLA_R5**	30434350	85.54%	37.25	30.43	4.57	98.32	95.12	81.71
**INS_Control**	30145498	85.21%	37.25	30.15	4.52	98.37	95.21	80.93
**INS_KETAMINE**	30601914	85.16%	37.25	30.6	4.59	98.39	95.31	82.16
**INS_R1**	29469980	85.58%	35.63	29.47	4.42	98.41	95.34	82.72
**INS_R5**	30199728	84.56%	37.25	30.2	4.53	98.3	95.05	81.08
**PVT_Control**	30033452	85.42%	37.25	30.03	4.51	98.39	95.28	80.63
**PVT_KETAMINE**	30524800	86.15%	37.25	30.52	4.58	98.38	95.19	81.95
**PVT_R1**	30454864	85.88%	37.25	30.45	4.57	98.4	95.28	81.76
**PVT_R5**	30270614	84.21%	37.25	30.27	4.54	98.25	94.93	81.27
