# Supplementary material for: Transcriptional changes in response to ketamine ester-analogs SN 35210 and SN 35563 in the rat brain
Source: BMC Genomics. 2019 Apr 11;20:281. doi: 10.1186/s12864-019-5649-6 (PMC6458767; doi:10.1186/s12864-019-5649-6)
Supplement: Supplementary file 4 — qPCR data. qPCR data for tested changes in gene expression. (DOCX 13 kb) [file 12864_2019_5649_MOESM4_ESM.docx]

**Supplementary data - qPCR**

**Quantitative PCR (qPCR) confirms patterns of change seen in transcriptome data**

Total RNA was extracted from a ~10mg tissue from each brain region from each of the animals using Trizol, followed by a kit-based RNA extraction protocol (Zymo RNA). cDNA was made from 1μg of total RNA using iScript™ RT Supermix (Biorad, NZ) and bought to 100ul with RNase-free water before storage at -20°C until use. To validate the deep sequencing results, qPCR was carried out for four transcripts on a MIC qPCR instrument (BioMolecular systems, Australia). Reaction reagents included HotFirePol Taq polymerase (Solis Biodyn, Estonia), 2.5mM MgCl2 , 200μM dNTPs, 1μM of each of forward and reverse oligonucleotide primers (sequences in supplementary table S1). 1ul of the diluted cDNA was used in every reaction. Primers were designed using NCBI PrimerBlast http://www.ncbi.nlm.nih.gov/tools/primer-blast and flank one or more introns where this design was possible. The analysis used normalisation of levels of three unchanging housekeeping genes (ACTB, UBC and B2M).

| **Gene name** | **Symbol** | **Primer sequence** | **Tm (°C)** | **Product**  **length** |
| --- | --- | --- | --- | --- |
| Glutamate Ionotropic Receptor AMPA Type Subunit 2 | GRIA2 | F. CAGCAGATTTAGCCCCTACG  R. CACACACCTCCAACAATGCG | 62.6  64.3 | 186 |
| Synaptopodin | SYNPO | F. TGGGCACCGGAGATTTCATC  R. TTCGAATCTTTGCCACTTGC | 64.8  62.1 | 111 |
| β-actin | ACTB | F. CTGAACCCTAAGGCCAACC  R. GTACGACCAGAGGCATACAGG | 62.7  64.0 | 116 |
| β-2-microglobulin | B2M | F. GTCACCTGGGACCGAGACAT  R. AGAAGATGGTGTGCTCATTGC | 65.8  63.5 | 138 |
| Ubiquitin C | UBC | F. CACCAAGAAGGTCAAACAGGA  R. AAGACACCTCCCCATCAAACC | 62.9  64.7 | 102 |

**Supplementary table S1.** Table showing PCR oligonucleotide primers used in qPCR to validate the patterns in the transcriptome sequencing.

The qPCR validation of transcriptome data for gene transcripts (Glutamate Ionotropic Receptor AMPA Type Subunit 2: GRIA2, and Synaptopodin: SYNPO) supported the patterns of changes seen in the transcriptome (Figure 5). SYNPO expression was confirmed as highly upregulated in BLA with R5.

***Insert supplementary figure 2 near here.***

**Supplementary Figure 1.** Graphs showing QPCR data for regions tested. Genes are (A) Synaptopodin (SYNPO) and (B) Glutamate Ionotropic Receptor AMPA Type Subunit 2 (GRIA2) (n=5 animals per treatment). * p<0.01, ** p<0.001. Red dotted line represents level in control/vehicle treated animals. Error bars are coefficient of variation (CV) for fold change data.
